# Supplementary material for: A Critical View of the Application of the APEX Software (Aqueous Photochemistry of Environmentally-Occurring Xenobiotics) to Predict Photoreaction Kinetics in Surface Freshwaters
Source: Molecules. 2019 Dec 18;25(1):9. doi: 10.3390/molecules25010009 (PMC7017383; doi:10.3390/molecules25010009)
Supplement: Supplementary file 1 [file molecules-25-00009-s001.zip › 3.2.4_gcc-4.4.0.zip!/3.2.4_gcc-4.4.0/3.2.4_gcc-4.4.0/doc/octave/html/interpreter/Coding-Tips.html]

Coding Tips - Untitled


Next: Comment Tips,
Previous: Style Tips,
Up: Tips and Standards


---

### C.2 Tips for Making Code Run Faster.

Here are some ways of improving the execution speed of Octave programs.

- Vectorize loops. For instance, rather than

  ```
            for i = 1:n-1
              a(i) = b(i+1) - b(i);
            endfor
  ```

  write

  ```
            a = b(2:n) - b(1:n-1);
  ```

  This is especially important for loops with "cheap" bodies. Often it suffices to vectorize
  just the innermost loop to get acceptable performance. A general rule of thumb is that the
  "order" of the vectorized body should be greater or equal to the "order" of the enclosing loop.- Use built-in and library functions if possible. Built-in and compiled functions are very fast.
    Even with a m-file library function, chances are good that it is already optimized, or will be
    optimized more in a future release.- Avoid computing costly intermediate results multiple times. Octave currently
      does not eliminate common subexpressions.- Be aware of lazy copies (copy-on-write). When a copy of an object
        is created, the data is not immediately copied, but rather shared. The actual
        copying is postponed until the copied data needs to be modified. For example:

        ```
                  a = zeros (1000); # create a 1000x1000 matrix
                  b = a; # no copying done here
                  b(1) = 1; # copying done here
        ```

        Lazy copying applies to whole Octave objects such as matrices, cells, struct,
        and also individual cell or struct elements (not array elements).

        Additionally, index expressions also use lazy copying when Octave can determine
        that the indexed portion is contiguous in memory. For example:

        ```
                  a = zeros (1000); # create a 1000x1000 matrix
                  b = a(:,10:100); # no copying done here
                  b = a(10:100,:); # copying done here
        ```

        This applies to arrays (matrices), cell arrays, and structs indexed using ().
        Index expressions generating cs-lists can also benefit of shallow copying
        in some cases. In particular, when a is a struct array, expressions like
        `{a.x}, {a(:,2).x}` will use lazy copying, so that data can be shared
        between a struct array and a cell array.

        Most indexing expressions do not live longer than their `parent' objects.
        In rare cases, however, a lazily copied slice outlasts its parent, in which
        case it becomes orphaned, still occupying unnecessarily more memory than needed.
        To provide a remedy working in most real cases,
        Octave checks for orphaned lazy slices at certain situations, when a value
        is stored into a "permanent" location, such as a named variable or cell or
        struct element, and possibly economizes them. For example

        ```
                  a = zeros (1000); # create a 1000x1000 matrix
                  b = a(:,10:100); # lazy slice
                  a = []; # the original a array is still allocated
                  c{1} = b; # b is reallocated at this point
        ```

        - Avoid deep recursion. Function calls to m-file functions carry a relatively significant overhead,
          so rewriting a recursion as a loop often helps. Also, note that the maximum level of recursion is
          limited.- Avoid resizing matrices unnecessarily. When building a single result
            matrix from a series of calculations, set the size of the result matrix
            first, then insert values into it. Write

            ```
                      result = zeros (big_n, big_m)
                      for i = over:and_over
                        r1 = ...
                        r2 = ...
                        result (r1, r2) = new_value ();
                      endfor
            ```

            instead of

            ```
                      result = [];
                      for i = ever:and_ever
                        result = [ result, new_value() ];
                      endfor
            ```

            Sometimes the number of items can't be computed in advance, and stack-like operations
            are needed. When elements are being repeatedly inserted at/removed from the end of an
            array, Octave detects it as stack usage and attempts to use a smarter memory management
            strategy preallocating the array in bigger chunks. Likewise works for cell and
            struct arrays.

            ```
                      a = [];
                      while (condition)
                        ...
                        a(end+1) = value; # "push" operation
                        ...
                        a(end) = []; # "pop" operation
                        ...
                      endwhile
            ```

            - Use `cellfun` intelligently. The `cellfun` function is a useful tool
              for avoiding loops. See Processing Data in Cell Arrays.
              `cellfun` is often use with anonymous function handles; however, calling
              an anonymous function involves an overhead quite comparable to the overhead
              of an m-file function. Passing a handle to a built-in function is faster,
              because the interpreter is not involved in the internal loop. For example:

              ```
                        a = {...}
                        v = cellfun (@(x) det(x), a); # compute determinants
                        v = cellfun (@det, a); # faster
              ```

              - Avoid calling `eval` or `feval` excessively, because
                they require Octave to parse input or look up the name of a function in
                the symbol table.

                If you are using `eval` as an exception handling mechanism and not
                because you need to execute some arbitrary text, use the `try`
                statement instead. See The `try` Statement.- If you are calling lots of functions but none of them will need to
                  change during your run, set the variable
                  `ignore_function_time_stamp` to `"all"` so that Octave doesn't
                  waste a lot of time checking to see if you have updated your function
                  files.
